# Breaking barriers in breast cancer therapy

**DOI:** 10.1002/1878-0261.13532

**Published:** 2023-10-03

**Authors:** Siddhi Maniyar, Ioannis Tsagakis

**Affiliations:** ^1^ Molecular Oncology Editorial Office Heidelberg Germany; ^2^ Molecular Oncology Editorial Office Cambridge UK

## Abstract

To honour Breast Cancer Awareness Month, we highlighted recent studies from the October issue focussing on overcoming therapy resistance in breast cancer. Wei et al. found that KH‐3 could counter the oncogenic effects of HuR, a regulator of docetaxel resistance in triple‐negative breast cancer (TNBC). Blasquez et al. showed the efficacy of ebselen oxide in repressing HER2, slowing down HER2‐positive breast tumour progression.

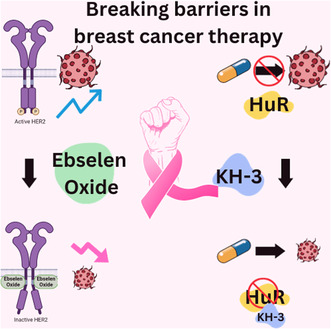

October is Breast Cancer Awareness Month, a time to acknowledge breast cancer and focus on educating those concerned about the disease and campaign to raise awareness about the impact of breast cancer. Under this prism, the October issue of *Molecular Oncology* includes several studies aimed at deepening our understanding of various aspects of breast cancer, including its mutational landscape, potential therapy targets, treatment effectiveness, response to treatment, prognostic markers and molecular mechanisms of advanced breast cancer.

Breast cancer encompasses a wide range of diseases with multiple subtypes. Among these, the worst survival is seen in patients with triple‐negative breast cancer (TNBC). Triple‐negative breast cancer (TNBC) is characterised by the absence of oestrogen receptor negative (ER^−^), progesterone receptor negative (PR^−^) and human epidermal growth factor receptor 2 negative (HER2^−^) and is associated with a 77% survival rate. Because each patient's genome carries a unique set of mutations, the efficacy of treatment can vary significantly from one individual to another. These mutations may also contribute to therapy resistance. Overcoming this resistance is the primary goal of recent efforts aimed at bringing more effective treatments for breast cancer to clinical settings.

In a study published in the October Issue of *Molecular Oncology*, the authors investigated TNBC cells resistant to a chemotherapy drug, docetaxel [1]. To better understand how to bypass resistance, they used a small molecule drug called KH‐3, which inhibits the function of the Hu antigen R (HuR) protein. This protein binds target mRNAs to stabilise them and increase their translation. It has documented roles in contributing to cancer progression by associating with mRNAs, the proteins of which, support cell growth and metastasis.

They found that KH‐3 could reduce cell growth in cells that were sensitive or resistant to docetaxel. More specifically, in docetaxel‐resistant cells, combined treatment of docetaxel and KH‐3 enhanced apoptosis. To elucidate how KH‐3 could overcome therapy resistance, the authors profiled gene expression in the two cell lines and found differences in the levels of BCL2 (the target of docetaxel) and CTNNB1 (β‐catenin).

KH‐3 seemed to lower the levels of β‐catenin, which was linked to a decrease in BCL2. Additionally, the researchers confirmed that KH‐3 could induce cell apoptosis. Therefore, KH‐3 appeared to sensitise TNBC cells to docetaxel by downregulating β‐catenin and BCL2. In experiments with mice that had docetaxel‐resistant tumours, KH‐3 treatment alone or in combination with docetaxel slowed down tumour growth. Additionally, mice treated with KH‐3 alone lived for 3 days longer on average, and those receiving both treatments lived for 7 days longer compared with mice receiving a control substance (DMSO).

Another study by Blasquez et al. [2] explored new therapeutic avenues against HER2‐positive cancers. HER2, a tyrosine kinase receptor, is overexpressed in a quarter of primary human breast cancers, driving aggressive tumour growth and challenging conventional treatments. While HER2‐targeted therapies have revolutionised patient outcomes, the emergence of resistance mechanisms and accompanying toxicities has underscored the urgency for innovative interventions.

This investigation shed light on the role of the ezrin/radixin/moesin (ERM) family of proteins, which, when present in normal cells, interact directly with HER2 to maintain it in a repressed state. However, in HER2‐overexpressing tumours, the scarcity of moesin results in an abnormal activation of HER2, contributing to cancer aggressiveness. Leveraging this, researchers aimed to discover a molecule capable of mimicking the modulatory role of moesin. Strikingly, they identified ebselen oxide through a meticulous screening process, showcasing its potential as an allosteric inhibitor capable of disrupting HER2's aberrant activation in HER2‐overexpressing tumours.

The study demonstrated ebselen oxide's remarkable potency in repressing not only HER2 but also mutated and truncated oncogenic variants of the HER2 receptor, which had hitherto proven resistant to prevailing therapies. Experiments in mice models demonstrated its remarkable ability to halt HER2‐positive breast tumour progression, reassuring its potential as a formidable therapeutic candidate. Furthermore, ebselen oxide's synergistic effects when combined with existing anti‐HER2 agents open possibilities for combinatorial therapies that could potentially elevate treatment outcomes.

These discoveries represent key advances in the journey towards overcoming resistance and hold profound clinical implications in treating treatment‐resistance breast cancers.
